# Digital PCR for high sensitivity viral detection in false-negative SARS-CoV-2 patients

**DOI:** 10.1038/s41598-021-83723-x

**Published:** 2021-02-22

**Authors:** Paolo Poggio, Paola Songia, Chiara Vavassori, Veronica Ricci, Cristina Banfi, Silvia Stella Barbieri, Gloria Garoffolo, Veronika A. Myasoedova, Luca Piacentini, Angela Raucci, Alessandro Scopece, Elena Sommariva, Maria Cristina Vinci, Davide Carcione, Maria Luisa Biondi, Maria Elisabetta Mancini, Alberto Formenti, Daniele Andreini, Emilio M. Assanelli, Piergiuseppe Agostoni, Marina Camera, Gualtiero I. Colombo, Maurizio Pesce

**Affiliations:** 1 Centro Cardiologico Monzino, IRCCS, Via Carlo Parea, 4, 20138 Milan, Italy; 2 Department of Clinical Sciences and Community Sciences, University of Milan, Milan, Italy; 3 Department of Clinical Medicine and Surgery, University Federico II, Naples, Italy; 4 Department of Pharmaceutical Sciences, University of Milan, Milan, Italy; 5 Unità di Ingegneria Tissutale Cardiovascolare, Centro Cardiologico Monzino, IRCCS, Via Carlo Parea, 4, 20138 Milan, Italy

**Keywords:** Laboratory techniques and procedures, Viral infection

## Abstract

Patients requiring diagnostic testing for coronavirus disease 2019 (COVID-19) are routinely assessed by reverse-transcription quantitative polymerase chain reaction (RT-qPCR) amplification of Sars-CoV-2 virus RNA extracted from oro/nasopharyngeal swabs. Despite the good specificity of the assays certified for SARS-CoV-2 molecular detection, and a theoretical sensitivity of few viral gene copies per reaction, a relatively high rate of false negatives continues to be reported. This is an important challenge in the management of patients on hospital admission and for correct monitoring of the infectivity after the acute phase. In the present report, we show that the use of digital PCR, a high sensitivity method to detect low amplicon numbers, allowed us to correctly detecting infection in swab material in a significant number of false negatives. We show that the implementation of digital PCR methods in the diagnostic assessment of COVID-19 could resolve, at least in part, this timely issue.

## Introduction

As outlined in several reports^1–3^, the problem of false-negative detection of Sars-CoV-2 in oro/nasopharyngeal swabs material continues to have a major impact on the management of the patients. Despite as of today exist more than 100 different certified tests for viral nucleic acid amplification, mainly based on reverse transcriptase quantitative polymerase chain reaction (RT-qPCR)^4,5^, and further nucleic acid^6,7^ or even protein detection systems—e.g. MALDI-mass spectrometry^8^—are coming into the scenario, this problem has not been solved yet. In the case of negative results of the molecular diagnostic method, other parameters are taken into account to diagnose COVID-19, such as the typical radiologic appearance of the lungs as detected by a Computer Tomography scan (CT-scan)^9^. The use of nucleic acid amplification methods such as the droplet digital PCR (ddPCR) has been proposed to resolve this problem, enabling detection of SARS-CoV-2 virus in superior airways with a theoretical sensitivity of 1 copy/reaction^10,11^. The present work was designed to retrospectively assess with a chip-based digital PCR platform (herewith-named dPCR) the presence of low viral titers in the RNA extracted from swab samples of patients with radiologic features of COVID-19 pneumonia testing negative to conventional diagnostic RT-qPCR.

## Materials and methods

### Ethical information

The present study was conducted after notification to the competent ethical committee, and conforming to the current laws on the management of patient personal data, and to the principles outlined in the Declaration of Helsinki of 1964. The approval of an informed consent was waived according to a specific FAQ (*“Data processing in clinical trials and medical research in the context of the COVID-19 health emergency”*—article 3), published by the Italian Data Protection Authority to rule the use of patients material for experimental studies on COVID-19 (See https://www.garanteprivacy.it/temi/coronavirus/faq#English) for more information.

### Patient characteristics

This is a retrospective, observational cohort study. We included in the study 64 consecutive patients admitted to our Hospital between February 27 and April 29, 2020, with clinical symptoms of pneumonia and COVID-19-typical chest computed tomography (CT) features^12^. Patients without COVID-19 imaging features or with contraindication to CT were excluded.

### Chest computed tomography

CT-scans were performed using a 256-slices CT scanner (Revolution CT; GE Healthcare, Milwaukee, WI). No contrast media were administered to the patients. CTs were recorded as positive in the presence of viral pneumonia imaging features^12^. In particular, we assessed ground-glass opacities (GGO), distribution, consolidations, multilobar involvement, crazy paving, air bronchogram, and the amount of infected lung using multiplane reconstructions.

### Diagnostic RT-qPCR assay

Our microbiological diagnostic laboratory adopts the GeneFinder COVID-19 Plus RealAmp Kit, a One-Step Reverse Transcription Real-Time PCR (RT-PCR) Kit designed to detect the presence of SARS-CoV-2 by quantitative amplification of the *RdRp*, *E,* and *N* genes (Elitech). The GeneFinder™ COVID-19 PLUS RealAmp Kit is used with RNA extracted in a robotized 12-channel RNA extraction/ RT-PCR amplification platform (ELITe InGenius SP200) using nasopharyngeal swabs material (NPS) collected in UTM medium (3 mL) within 72 h after collection under appropriate storage conditions (+ 2 to + 8 °C).

The diagnostic runs are performed as follows: after thawing working solutions (COVID-19 PLUS Reaction Mixture and COVID-19 PLUS Probe Mixture), an RT-PCR master mix is prepared and stored at 4 °C in a thermally controlled rack in the platform. In parallel, an operator manually dispenses 0.2 mL of the NPS material in dedicated sample tubes installed in the sample line in the platform. The Ingenius instrument is set to perform RNA extraction using a bead-based protocol and produces a 100 μL total volume of eluted RNA, which is automatically transferred to recovery tubes and immediately used for RT-PCR. The eluted RNAs produced by the platform were immediately frozen at the end of each diagnostic run.

The protocol for diagnostic SARS-CoV-2 genes detection is performed by automatic dispensing 5 μL of the RNA eluate together with 15 μL of the RT-PCR Master Mixture into preloaded PCR tubes, followed by a 45 cycles amplification program. The expected detection targets consist of viral *RdRp*, *N*, and *E* RNAs and *RNAseP* cellular mRNA (as an internal control, IC) with these respective fluorophores: FAM, VIC, Texas Red, and Cy5. A sample is considered positive when one or more of the genes are detected below 40 threshold cycles (Ct) or below 35 Ct for IC. The specificity of the procedure is declared to be 100%, with an analytical sensitivity of 10 copies/test for each gene by the Manufacturer (https://www.elitechgroup.com/documents?search=SARS).

### ACE2 detection by RT-PCR

Detection of the *ACE2* mRNA was performed using 4 μL of the eluted RNAs produced by the automated platform. The RT-qPCR analysis was performed with an ABI Prism 7900 HT (Applied Biosystems) with the FAM chemistry (Thermo Fisher). As a PCR reaction mix, we used the TaqMan fast one-step Master Mix (Thermo Fischer), the ACE2 primers (Hs01085333_m; Thermo Fischer), the RPLP0 primers (Hs99999902_m1; Thermo Fischer), or the RPL32 primers (Hs00851655_g1; Thermo Fischer). Reverse transcription (RT) was performed at 50 °C for 5 min, followed by RT inactivation/initial denaturation at 95 °C for 20 s. PCR cycling consisted of a denaturation step at 95 °C for 5 s followed by annealing/extension at 60 °C for 30 s (45 cycles). For ACE2 as well as two reference genes (RPLP0 and RPL32), the cycle threshold (Ct) value was determined and the dCt value was calculated (target Ct – mean reference gene Ct).

### Digital PCR assay

To proceed with a direct comparison of the efficiency of digital vs. conventional RT-qPCR assay, dPCR was performed using 5 μL of the eluted RNAs collected from the automated platform performing the RT-qPCR diagnostic assay, and appropriately stored at − 80 °C. The dPCR analysis was performed with a QuantStudio 3D Digital PCR System platform consisting of a QuantStudio 3D Instrument, a Dual Flat Block GeneAmp PCR System 9700, and a QuantStudio 3D Digital PCR Chip Loader (all from Thermo Fisher). As a PCR reaction mix, we used the TaqMan fast Virus one-step Master Mix (Thermo Fischer). We used primers and probes authorized from the Center for Disease Control and Prevention (CDC) for the SARS-CoV-2 *N* gene (*N1* and *N2*) (referred in^7^). As a positive control, we included primers specific for the *RNAseP* (a non-viral transcript); water was added as a negative control. Reverse transcription (RT) was performed at 50 °C for 10 min, followed by RT inactivation/initial denaturation at 96 °C for 5 min. PCR cycling consisted of a denaturation step at 98 °C for 30 s followed by annealing/extension at 56 °C for 1 min (40 cycles), and a final extension at 60 °C for 5 min. Data analysis was performed with the online version of the QuantStudio 3D AnalysisSuite (Thermo Fisher Cloud). The expected detection targets consist of the viral *N* (nucleocapsid protein gene) and cellular *RNAseP* (as an internal control, IC) mRNAs with FAM fluorophore. The lower limit of detection (LOD) of the dPCR method was set by adding three standard deviations to the mean of the background signal achieved in three PCR amplifications with water (0.149 ± 0.0132 and 0.163 ± 0.008 copies/µL, for *N1* and *N2* primers, respectively). This limit corresponded to ~ 2.2 copies/mL per each primers set.

### Statistical analysis

Clinical and CT characteristics are expressed as counts and proportions in the case of categorical variables. Most clinical continuous variables did not pass the D'Agostino-Pearson omnibus normality test and, thus, are reported as medians and interquartile ranges. No imputation was made for missing data points. Categorical variables were compared by the χ^2^ test or Fisher’s exact test, as needed. Group comparisons for continuous data were performed with the Kruskal–Wallis test, followed by Dwass–Steel–Critchlow–Fligner pairwise comparisons if the previous test was significant. We directly compared the sensitivity of dPCR with diagnostic RT-qPCR assay by computing the Pearson’s correlation coefficient (r_P_) between log_10_ copies/mL of N1 or N2 amplicons detected by the dPCR test and the Ct values of the *N* gene detected by the diagnostic test. The level of statistical significance was set at *P* < 0.05. Statistical analysis was performed using the *jamovi* software version 1.2.17 (https://www.jamovi.org).

## Results

**Figure 1:**
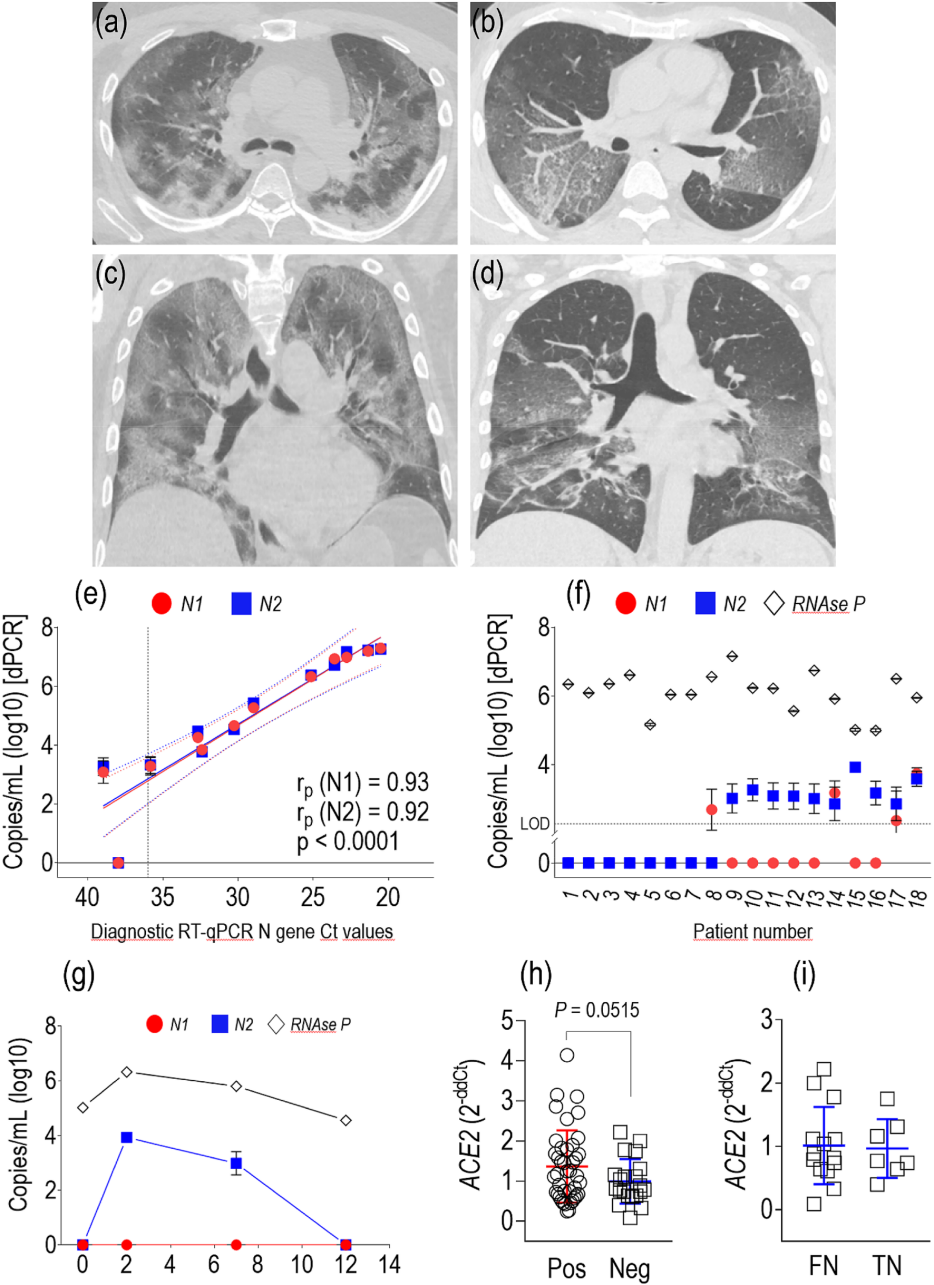
(**a**–**d**) Chest CT axial and coronal projections at the level of tracheal bifurcation in a SARS-CoV-2 positive patient (**a**,**c**) and patient #15 (**b**,**d**), admitted to our Center. (**e**) Correlation between the copies of *N1*/*N2* amplicons (expressed as log_10_ copies/mL) detected by dPCR and the Ct values of the *N* gene by the single primer present in the diagnostic test. It is evident a better correlation of the data below ~ 36 Ct (evidenced by the dotted line in the graph). (**f**) Individual results of digital PCR analysis of the 18 patients scoring negative in the conventional diagnostic test. In patients 1–7, the digital PCR test was unable to detect the virus in the diagnostic eluted RNA. Patients 8–18 exhibited a low copy number of *N1*, *N2* amplicons, or both. The line labeled with LOD indicates the lower detection limit of ~ 2.2 copies/mL, calculated as described in materials and methods section. (**g**) Patient #15 was tested in four consecutive swabbing procedures and was invariantly negative wih the diagnostic assay. Re-testing by digital PCR of the eluted RNA showed a clear SARS-CoV-2 positivity by *N2* sequence amplification. (**h**) The *ACE2* mRNA was detected by conventional RT-PCR using the eluate RNA extracted from the swab material. ACE2 expression levels showed a decreased trend in negative vs. positive patients (*P* = 0.0515 by *t* test). (**i**) no differences were observed in the levels of *ACE2* expression in the true-negative (TN) and false-negative (FN) subjects. In panels (**h**) and (**i**) data are reported as average ± standard deviation.

Patient population characteristics, as well as CT findings, are listed in Table 1. The overall median age was 67.0 (55.3–77.3) years with 39 males and 25 females. In the overall population, the prevalent symptoms on admission were fatigue (44 out of 64 patients, 68.8%), dyspnea (41 out of 64, 64.1%), and fever (34 out of 64, 53.1%). On admission, lymphocytopenia was present in 60.9% of the patients (39 out of 64). Most of the patients had elevated levels of C-reactive protein (PCR) and B-type natriuretic peptide (BNP). Only 7 patients presented with moderate acute respiratory distress syndrome (PaO2/FiO2 > 100 mmHg and ≤ 200 mmHg). Most of the patients had typical COVID-19 CT features^12^, such as GGO and consolidations with peripheral distribution and multi-lobar involvement with vascular enlargement (Fig. 1a–d). In this cohort, we retrospectively identified a group of 18 subjects who tested negative for SARS-CoV-2 with the conventional diagnostic RT-qPCR assay performed on oro/nasopharyngeal swab material. These patients were screened once (n = 5) or twice (n = 12) at variable time intervals (1–25 days), and never exhibited conversion to positivity. One patient (#15 in Table S1) was assessed four times in 12 days, always testing negative.

**Table 1:** Demographic and clinical characteristics of the patients.

Characteristic	Total (*n* = 64)	RT-qPCR^neg^ dPCR^neg^ (*n* = 7)	RT-qPCR^neg^ dPCR^pos^ (*n* = 11)	RT-PCR^Pos^ (*n* = 46)	*P*
Age, years	67.0 (55.3–77.3)	73.0 (62.5–81.0)	71.0 (52.0–81.5)	66.5 (56.0–76.5)	0.5327
Male sex—*n* (%)	39 (60.9)	4 (57.1)	7 (63.6)	28 (60.9)	0.9627
BMI, kg/m^2^	25.9 (23.8–29.6)	27.6 (26.3–29.0)	28.7 (24.8–32.2)	25.6 (23.7–28.5)	0.5276
**Smoking habit—n (%)**					
Never	54 (84.4)	5 (71.4)	10 (90.9)	39 (84.8)	0.7611
Former	9 (14.1)	2 (28.6)	1 (9.1)	6 (13.0)	
Current	1 (1.6)	0 (0.0)	0 (0.0)	1 (2.2)	
**Symptoms on admission—n (%)**					
Fever	34 (53.1)	1 (14.3)	7 (63.6)	26 (56.5)	0.0845
Dry cough	26 (40.6)	3 (42.9)	2 (18.2)	21 (45.7)	0.2474
Shortness of breath	41 (64.1)	6 (85.7)	8 (72.7)	27 (58.7)	0.3073
Chest pain	14 (21.9)	3 (42.9)	2 (18.2)	9 (19.6)	0.3616
Fatigue	44 (68.8)	5 (71.4)	7 (63.6)	32 (69.6)	0.9178
Muscle or joint pain	23 (35.9)	1 (14.3)	4 (36.4)	18 (39.1)	0.4427
Nausea or vomiting	5 (7.8)	0 (0.0)	2 (18.2)	3 (6.5)	0.3101
Diarrhea	6 (9.4)	0 (0.0)	2 (18.2)	4 (8.7)	0.4162
**Comorbidities—n (%)**					
COPD or asthma	4 (6.3)	1 (14.3)	3 (27.3)	0 (0.0)	**0.0023**^**a**^
Hypertension	38 (59.4)	4 (57.1)	8 (72.7)	26 (56.5)	0.6118
Coronary artery disease	31 (48.4)	3 (42.9)	7 (63.6)	21 (45.7)	0.5359
Cerebrovascular disease	4 (6.3)	1 (14.3)	1 (9.1)	2 (4.3)	0.5469
Diabetes	17 (26.6)	0 (0.0)	3 (27.3)	14 (30.4)	0.2359
Hyperlipidemia	23 (35.9)	2 (28.6)	4 (36.4)	17 (37.0)	0.9109
Chronic kidney disease	9 (14.1)	1 (14.3)	3 (27.3)	5 (10.9)	0.3722
**Laboratory values**					
Hemoglobin, g/dL	12.4 (11.5–14.2)	11.5 (9.8–12.7)	11.6 (10.9–13.6)	12.8 (11.8–14.5)	0.1878
White blood cells, 10^3^/µL	7.50 (5.10–10.60)	11.00 (8.90–16.20)	7.90 (6.50–10.30)	6.80 (4.93–9.47)	0.0506
Neutrophils, 10^3^/µL	5.07 (3.48–8.55)	9.36 (5.63–15.10)	5.25 (4.20–9.46)	4.26 (3.45–7.43)	0.1041
Lymphocytes, 10^3^/µL	1.40 (0.90–1.70)	1.30 (0.90–2.40)	1.60 (0.90–1.80)	1.40 (0.93–1.60)	0.8784
Monocytes, 10^3^/µL	0.60 (0.48–0.90)	0.90 (0.65–1.00)	0.80 (0.35–0.85)	0.60 (0.43–0.80)	0.1627
Platelets, 10^3^/µL	222 (156–292)	262 (226–293)	209 (168–272)	214 (154–302)	0.4022
Prothrombin time (INR)	1.17 (1.12–1.29)	1.53 (1.21–1.63)	1.17 (1.12–1.27)	1.17 (1.11–1.26)	0.1944
aPTT (ratio)	1.23 (1.13–1.34)	1.13 (0.94–1.26)	1.19 (1.12–1.23)	1.24 (1.15–1.35)	0.3638
eGFR, mL/min/1.73 m^2^	72.5 (42.5–91.3)	63.0 (36.0–76.0)	66.0 (34.0–77.5)	83.5 (56.8–94.5)	0.0724
Blood glucose, mg/dL	123 (106–153)	119 (112–138)	125 (115–162)	122 (102–151)	0.8463
ALT, IU/L	30.5 (20.8–44.3)	36 (19–61.5)	41 (28–55)	30.0 (21.0–40.0)	0.3103
AST, IU/L	28.0 (17.0–52.0)	36 (18–69)	42 (29.5–45.5)	26.5 (16.0–50.3)	0.4761
BNP, pg/mL	182.0 (27.5–667.0)	1564 (740–2271)	82.6 (27.9–683)	130.0 (25.9–424.0)	**0.0160**^**b**^
Procalcitonin, ng/mL	0.07 (0.03–0.16)	0.10 (0.03–0.43)	0.10 (0.04–0.16)	0.06 (0.03–0.12)	0.8031
CRP, mg/L	23.4 (7.6–72.5)	31.0 (9.6–106.0)	80.0 (20.9–151.0)	17.2 (6.1–49.5)	**0.0499**^**c**^
Lymphocytopenia—*n* (%)	39 (60.9)	4 (57.1)	7 (63.6)	28 (60.9)	0.9627
Increased D-dimer—*n* (%)	8 (12.5)	2 (28.6)	2 (18.2)	4 (8.7)	0.2744
PaO_2_/FiO_2_ ratio, mm Hg	302 (248–408)	300 (260–362)	245 (233–282)	310 (264–421)	0.1530
**Chest CT features—n (%)**					
Total infected lung volume, %	23.9 (12.2–41.0)	20.8 (13.6–31.4)	23.9 (22.8–46.2)	24.5 (12.1–41.4)	0.6779
GGO + consolidation	28 (43.8)	1 (14.3)	6 (54.5)	21 (45.7)	0.2168
Air bronchogram	17 (26.6)	0 (0.0)	4 (36.4)	13 (28.3)	0.2079
Vascular enlargement	45 (70.3)	4 (57.1)	10 (90.9)	31 (67.4)	0.2226
Crazy paving	23 (35.9)	1 (14.3)	3 (27.3)	19 (41.3)	0.3073
Peripheral distribution	46 (71.9)	2 (28.6)	7 (63.6)	37 (80.4)	**0.0141**^**d**^
Multilobar involvement	56 (87.5)	7 (100)	11 (100)	39 (84.8)	0.2149
**Outcomes—n (%)**					
Mechanical ventilation	23 (35.9)	2 (28.6)	4 (36.4)	17 (37.0)	0.9109
Death	6 (9.4)	1 (14.3)	1 (9.1)	4 (8.7)	0.8937

Data are reported as median and interquartile range (Q1-Q3) for continuous variables and count and percentage for categorical variables.

*BMI* body mass index, *COPD* chronic obstructive pulmonary disease, *INR* international normalized ratio, *aPTT* activated partial thromboplastin time, *eGFR* estimated glomerular filtration rate, *ALT* alanine aminotransferase, *AST* aspartate aminotransferase, *BNP* B-type natriuretic peptide, *CRP* C-reactive protein, *PaO*_*2*_ arterial partial pressure of oxygen, *FiO*_*2*_ fraction of inspired oxygen, *CT* computed tomography, *GGO* ground-glass opacity.

Post-hoc pairwise comparisons:

^a^ *P* = 0.0056 for false negatives vs. positives.

^b^ *P* = 0.0122 for true negatives vs. positives.

^c^ *P* = 0.0346 for false negatives vs. positives.

^d^ *P* = 0.0104 for true negatives vs. positives.

To reconcile the negative results of the diagnostics RT-qPCR with a diagnosis of COVID-19 pneumonia based on clinical evaluation and CT-scan, we re-tested the eluted RNAs produced by the diagnostic pipeline using dPCR and primers for the SARS-CoV-2 *N* gene sequence approved by the U.S. CDC (referred in^7^) (*N1* and *N2*; Figure S1). Method calibration was performed using eluted RNAs from positive patients: we found a good correlation between viral copy numbers detected with digital PCR and Ct values scored by diagnostic RT-qPCR, at least up to ~ 36 Ct, corresponding to ~ 1000 copies/mL (Fig. 1e), the lower copy number for reliable viral detection as demonstrated elsewhere^13^. The analysis revealed that 11 (61%) of the 18 RT-qPCR negative patients had a detectable number of SARS-CoV-2 copies, with either one or both primers’ sets (Fig. 1f) when tested with dPCR. Interestingly, patient #15 (invariantly negative with the RT-qPCR test) showed conversion from negativity to positivity and vice-versa with dPCR, with a peak in copy number in the second swab test (Fig. 1g).

We then analyzed whether there were differences between groups in the clinical, laboratory, and CT imaging characteristics when patients were stratified according to dPCR positivity for SARS-CoV-2. We distinguished patients into: (*i*) RT-qPCR^neg^/dPCR^neg^ (herewith referred as to ‘true negatives’; n = 7), when both the diagnostic test and the digital PCR test were negative. (*ii*) RT-qPCR^neg^/dPCR^pos^ (herewith referred as to ‘false negatives’; n = 11), when the diagnostic test was negative but the digital PCR test was positive. (*iii*) RT-qPCR^pos^ (herewith referred as to ‘positives’; n = 46), when samples were positive with the conventional diagnostic test. As shown in Table 1, we did not observe differences in the demographic characteristics and symptoms on admission between the three groups. Likewise, there were no differences in comorbidities, except for a higher prevalence of previous lung diseases (chronic obstructive pulmonary disease or asthma) in the false-negative group than in the positive group (27.3% vs. 0%, respectively; *P* = 0.0056). Blood count, coagulation tests, hepatic and renal functions, and blood glucose were similar among the groups. Conversely, BNP median plasma concentration was higher in true negatives than in positives (1546 [740–2271] vs*.* 130 [25.9–424], respectively; *P* = 0.0122) and CRP levels were more elevated in the false-negative than in the positive group (80 [20.9–151] vs. 17.2 [6.1–49.5]; *P* = 0.0346). Interestingly, there was a decreasing trend in white blood cell count, which dropped from a median of 11.0 × 10^3^/µL (8.9–16.2) in true negatives to 7.9 × 10^3^/µL (6.5–10.3) in false negatives, to 6.8 × 10^3^/µL (4.9–9.5) in positives, with a significant difference between false negatives and positives at post-hoc analysis (*P* = 0.037). Finally, there were no differences in chest CT features and outcomes among the three groups, except for a higher prevalence of peripherally distributed GGOs or consolidations in positive than in true negative patients (80.4% vs. 28.6%, respectively; *P* = 0.0104).

Recently, a single-cell RNA profiling of SARS-CoV-2/coronavirus-associated receptors and factors (SCARFs) expression has been performed in several human tissues including the nasal epithelium^14^. Since a relatively high variability in the expression of these receptors (e.g. ACE2) was reported, with possible implications for the severity of the infection in superior airways, we measured *ACE2* in the RNA contained in the swab eluates and correlated this level to the positive/negative status of our patients. As shown in Fig. 1h, the *ACE2* level exhibited a lower trend in negative subjects, with no discrimination between true and false negatives (Fig. 1i).

## Discussion

Our results show that in the cohort of subjects with COVID-19 pneumonia who scored negative with the diagnostic SARS-CoV-2 RT-qPCR (18/64), 11/64 (~ 17%) turned out to be false negatives with dPCR amplification, thus increasing the overall sensitivity of the virus molecular detection from ~ 72 to ~ 89%. This finding consolidates the utility of high-resolution amplification methods, as ‘second-level’ assays to detect SARS-CoV-2 infection in subjects with clear COVID-19 pneumonia and low viral replication in the superior airways^3,11,15^. Limitations in the use of dPCR still exist, considering that a number of subjects in our cohort (the true negatives; 7/64; ~ 11%) did not exhibit positive amplification of the viral *N* gene sequences even with this high sensitivity technique. Other factors such as the quality of the swabbing procedure and the reported absence of detectable viral replication in superior airways^2^ could account for the failure to detect SARS-CoV-2 in the RNA material of these subjects, even with the highest performance detection methods. A preventive strategy to minimize this problem is to perform more thorough and comprehensive material collection, e.g. by concentrating on multiple respiratory sites^16^, repeat tests at different times during the course of the illness, or test broncho-alveolar aspirate in addition to the superior airways material^17^.

Although we cannot provide an explanation for the different replication of the virus in superior airways and its potential relationships with alterations of laboratory markers and severity of the pathology^18^, we noticed a lower trend in the expression *ACE2* (one of the major SARS-CoV-2 receptors^19^) in patients with negative RT-qPCR diagnosis (Fig. 1h). If these trends were confirmed in studies with adequate sample size, it would provide additional criteria to diagnose patients with higher accuracy.

In summary, confirming previous reports, we show the superior performance of dPCR amplification for the diagnosis of SARS-CoV-2 infection in subjects with low viral titers in conventional swab tests. Furthermore, given the existence of multiple receptors and intracellular pathways involved in virus entry into cells^14^, this method seems useful to investigate the biological basis of low SARS-CoV-2 replication in the superior airways in a significant proportion of symptomatic COVID-19 patients, with benefits for the overall outbreak management.

## Supplementary Information

Supplementary Information.
