# Supplementary material for: Digital PCR for high sensitivity viral detection in false-negative SARS-CoV-2 patients
Source: Sci Rep. 2021 Feb 22;11:4310. doi: 10.1038/s41598-021-83723-x (PMC7900100; doi:10.1038/s41598-021-83723-x)

**Digital PCR for high sensitivity viral detection in false-negative SARS-CoV-2 patients**

Paolo Poggio^1,*^, Paola Songia^1,*^, Chiara Vavassori^1,2^, Veronica Ricci^1,3^, Cristina Banfi^1^, Silvia Stella Barbieri^1^, Gloria Garoffolo^1^, Veronika A. Myasoedova, Luca Piacentini^1^, Angela Raucci^1^, Alessandro Scopece^1^, Elena Sommariva^1^, Maria Cristina Vinci^1^, Davide Carcione^1^, Maria Luisa Biondi^1^, Maria Elisabetta Mancini^1^, Alberto Formenti^1^, Daniele Andreini^1,2^, Emilio M. Assanelli^1^, Piergiuseppe Agostoni^1,2^, Marina Camera^1,4^, Gualtiero I. Colombo^1,#^ and Maurizio Pesce^1,#^

**Supplementary table and figure**

**Table S1.** Results of dPCR analysis with the indication of the absolute copy number/µL detected by amplification with *N1* and *N2* primers pairs in eluted RNA of patients with negative detection of COVID-19 by conventional PCR, at each testing time intervals.

In 7 patients, digital PCR was unable to detect any viral copy at all testing times. In 11 subjects, digital PCR detected absolute copy numbers ranging from 0.24/µL (patient 17; first swab, *N1*) to 8.59 (patient 15; second swab, *N2*) per test. Note the trend found for *N2* amplification in patient #15, a subject tested four times and invariantly found negative by conventional test.

| Patient # | Days between consecutive tests (0 when patients were tested once) | *N1* (copies/µL) | | | | *N2* (copies/µL) | | | |
| --- | --- | --- | --- | --- | --- | --- | --- | --- | --- |
|  |  | **T1** | **T2** | **T3** | **T4** | **T1** | **T2** | **T3** | **T4** |
| 1 | 0 | 0 |  |  |  | 0 |  |  |  |
| 2 | 2 | 0 | 0 |  |  | 0 | 0 |  |  |
| 3 | 1 | 0 | 0 |  |  | 0 | 0 |  |  |
| 4 | 4 | 0 | 0 |  |  | 0 | 0 |  |  |
| 5 | 1 | 0 | 0 |  |  | 0 | 0 |  |  |
| 6 | 0 | 0 |  |  |  | 0 |  |  |  |
| 7 | 5 | 0 | 0 |  |  | 0 | 0 |  |  |
| 8 | 1 | 0.49 | 0 |  |  | 0 | 0 |  |  |
| 9 | 0 | 0 |  |  |  | 1.04 |  |  |  |
| 10 | 1 | 0 | 0 |  |  | 0 | 1.85 |  |  |
| 11 | 0 | 0 |  |  |  | 1.04 |  |  |  |
| 12 | 1 | 0 | 0 |  |  | 0 | 1.85 |  |  |
| 13 | 9 | 0 |  |  |  | 1.22 |  |  |  |
| 14 | 0 | 1.52 |  |  |  | 0.72 |  |  |  |
| 15 | 2, 5, 5 | 0 | 0 | 0 | 0 | 0 | 8.59 | 0.96 | 0 |
| 16 | 25 | 0 | 0 |  |  | 1.50 | 0.77 |  |  |
| 17 | 1 | 0.24 | 0 |  |  | 0.72 | 1.09 |  |  |
| 18 | 22 | 5.46 | 0 |  |  | 3.91 | 2.29 |  |  |

**Figure S1.** Representative images of the chip-based digital PCR amplification results on > 15.000 nano-wells in the FAM fluorescent channel of eluted RNAs from patients with high (Ct ≤ 20), low (20 ≤ Ct ≤ 40) or negative (Ct > 40) detection SARS-CoV-2 by diagnostic RT-qPCR Cts. Images in the left and the right panels represent, respectively, the amount and the distribution of the nCOV_1 and RNAseP amplicons (blue dots) in each chip and their FAM fluorescence intensity.


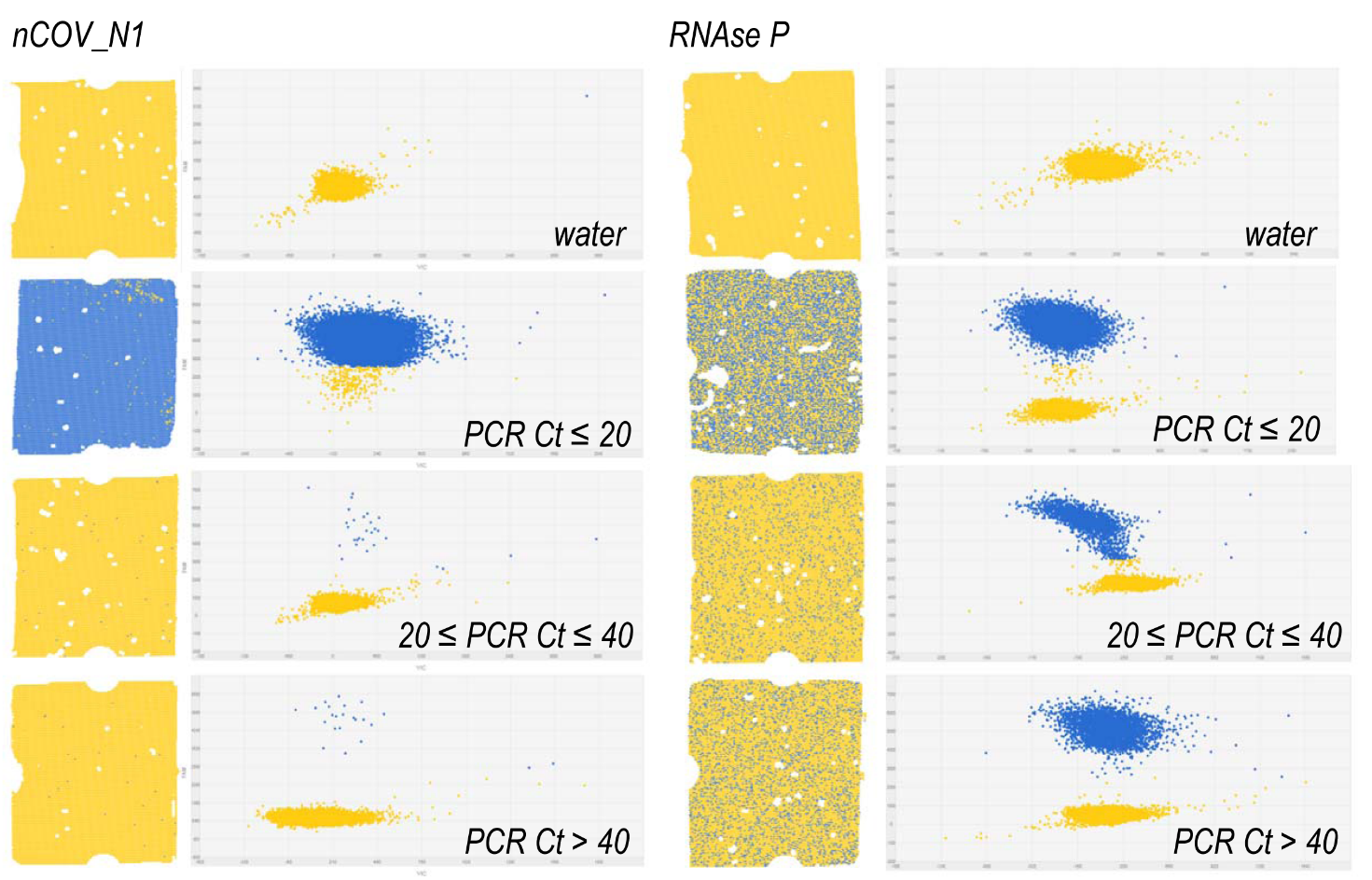

Supplement: Supplementary file 1 — Supplementary Information. [file 41598_2021_83723_MOESM1_ESM.docx]
